# The Specific Copper(II) Chelator TDMQ20 Is Efficient for the Treatment of Wilson’s Disease in Mice

**DOI:** 10.3390/pharmaceutics15122719

**Published:** 2023-12-02

**Authors:** Yingshan Zhu, Ying Tang, Lan Huang, Michel Nguyen, Yan Liu, Anne Robert, Bernard Meunier

**Affiliations:** 1 School of Chemical Engineering and Light Industry, Guangdong University of Technology (GDUT), Higher Education Mega Center, Guangzhou 510006, China; eshan041726@163.com (Y.Z.); yingtang0852@163.com (Y.T.); hannahhl@126.com (L.H.); 2 Laboratoire de Chimie de Coordination du CNRS, Inserm ERL 1289, 205 Route de Narbonne, CEDEX 4, 31077 Toulouse, France; michel.nguyen@lcc-toulouse.fr (M.N.); anne.robert@lcc-toulouse.fr (A.R.)

**Keywords:** copper chelator, liver, toxic milk mouse, Wilson’s disease

## Abstract

(1) Background: In patients with Wilson’s disease, the deficiency of the copper carrier ATP7B causes the accumulation of copper in the liver, brain and various other organs. Lifelong treatment is therefore mandatory, using copper chelators to increase the excretion of copper and to avoid life-threatening damage. The clinically used reference drug, D-penicillamine, exhibit numerous adverse effects, especially a frequent severe and irreversible neurological worsening, mainly due to its lack of metal selectivity; (2) Methods: A new tetradentate ligand based on an 8-aminoquinoline entity, named TDMQ20, which is highly selective for copper compared with other metal ions, is evaluated in “toxic milk” TX mice as an oral treatment of this Wilson’s disease murine model; (3) Results: The concentration of copper in the liver of “toxic milk” TX mice decreased and the fecal excretion of copper increased upon oral treatment with TDMQ20. Both effects are dose-dependent, and more pronounced than those of D-penicillamine; (4) Conclusions: The TDMQ20 copper chelator is more efficient than the reference drug D-penicillamine for the treatment of a Wilson’s disease murine model. Pharmacological data obtained with TDMQ20 on the TX mouse model strongly support the selection of this ligand as a drug candidate for this genetic disease.

## 1. Introduction

Wilson’s disease (WD) is a genetic disease caused by mutations on the Atp7b gene coding for a copper carrier protein in charge of the excretion of excess copper from the liver. This autosomal recessive disorder of copper metabolism concerns one in 30,000 people in the world. The ATP7B transporter is involved in incorporating copper in apoceruloplasmin, which is finally eliminated to bile and then to feces. The deficiency of this copper carrier causes the accumulation of copper in the liver, consequently being responsible for acute or chronic hepatitis and liver cirrhosis, potentially leading to fulminant hepatic failure. Copper is partially released in the bloodstream and slowly eliminated in urine. In advanced disease, neurological symptoms such as seizures or Parkinsonism typically appear. Psychiatric disease (depression, sleep disturbance, bipolar disorder, schizophrenia, etc.) is also frequently observed in WD patients [1,2]. Ocular manifestations are also frequent. As copper can accumulate in various organs, WD has been associated with various other symptoms, especially renal and cardiac pathologies as well as hypoparathyroidism and osteoarticular damages, factors correlated with a fatal prognosis. Lifelong treatment is therefore necessary, using copper chelators to increase the excretion of copper. One of the first-line drugs for WD treatment is currently D-penicillamine (DPA, 3-mercapto-D-valine) via oral administration at 750–1500 mg/day in adults. Several adverse effects of DPA have been identified with time: nephrotoxicity, aplasia, retinitis and neurological deterioration. Another drug used for WD is trientine (triethylene–tetramine), with oral doses ranging from 900 to 2700 mg/day in adults. Severe adverse effects are also observed with time: bone marrow depression, anemia, skin rash and hemorrhagic gastritis. These chelators are often associated with zinc salts in order to induce metallothionein synthesis and consequently to prevent copper intestinal absorption [3]. However, the limitations of treating a patient at the same time with a metal (zinc) and a chelating agent have been emphasized [4]. The efficacy of DPA in neurologic WD is only moderate (55% of improvement rate). Moreover, DPA and trientine exhibit numerous adverse effects that may be serious enough to require the discontinuation of treatment in approximately 30% of patients for DPA [5,6]. Both DPA and trientine induce a severe and irreversible neurological worsening in 10–50% of patients with previous neurological symptoms [7]. Therefore, there is a real medical need for a specific copper chelator that is able to efficiently regulate the copper excess, at lower doses than those used for DPA or trientine and with less side effects in the long term.

Besides a moderate efficacy, the main drawback of DPA and trientine is their lack of metal selectivity. In fact, both have been reported to coordinate with high affinity, a wide variety of metal ions, and oxidation states [8]. For example, DPA coordinates Cu^2+^ with log *K*_1_ = 16.5, but also Cu^+^ (log *K*_1_ = 19.5), Zn^2+^ (log *β*_2_ = 19.6) [8], and Fe^2+^, Fe^3+^, Co^2+^ and Co^3+^ [9], along with many other non-biological metals [8]. In addition, the structures of metal complexes of DPA can be diverse, including ternary complexes that involve other amino acids such as histidine or methionine [10,11]. A mixed valence cluster complex of Cu(I) and Cu(II) with formula [Cu^II^_6_Cu^I^_8_DPA_12_Cl]^5–^ has also been reported [12]. Moreover, the ability of DPA to act as a reductant due to its thiol functionality and to coordinate both Cu(II) and Cu(I) [or Fe(III) and Fe(II)] may confer DPA the capacity to trigger deleterious Fenton-like reactions and damage.

**Figure 1:**
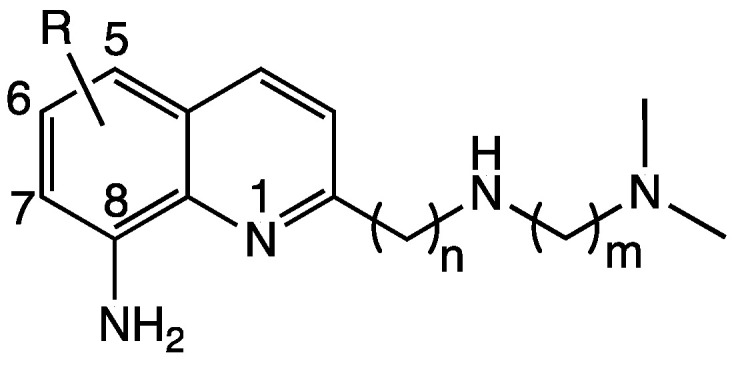
General formula of TDMQ ligands. For TDMQ20: R = 5,7-dichloro-, n = m = 2.

Our research group designed a new series of tetradentate ligands based on an 8-aminoquinoline entity, named TDMQ, that are highly selective for copper compared with other metal ions like zinc, and that are unable to accommodate Cu(I). One of these chelators, TDMQ20 (Figure 1), has qualified as a drug candidate for the treatment of Alzheimer’s disease [13,14,15,16]. Since TDMQ20 is safe for long-term oral administration [16] and has a high bioavailability [17], we decided to evaluate its activity as a drug candidate for the treatment of Wilson’s disease. In addition, because of the very weak affinity of TDMQ20 for zinc, it should be noted that this ligand will be compatible with the co-treatment using zinc acetate that is currently used in WD therapy to reduce the absorption of copper in the intestinal track [1].

The results provide evidence that TDMQ20 has a better pharmacological activity at lower doses via oral administration than D-penicillamine (DPA), improving the normal/non-pathological pathway of copper excretion in the toxic milk (TX) mouse model of Wilson’s disease.

## 2. Materials and Methods

### 2.1. Chemicals and Methods

All solvents and commercially available reagents were purchased from usual chemical suppliers, and were used without further purification. Cu(II) chloride or sulfate were used as sources of metal ions. Two lots of recombinant bovine erythrocytes, namely Cu,Zn-SOD expressed in E. coli (EC number 1.15.1.1, Sigma S9697-75KU, 4506 units/mg protein and Sigma S7571–75KU, 5236 units/mg protein) were used. The Cu,Zn-superoxide dismutase (SOD for short) colorimetric activity kit was from Sigma-Aldrich, Saint-Quentin-Fallavier, France (Ref. 19160-1KT-F). TDMQ20 was prepared according to Reference [14]. The solvents used for histology were purchased from Sinopharm Chemical Reagents Co., Ltd., Shanghai, China.

#### 2.1.1. Copper Dosage

The copper concentrations in the urine and feces, serum, liver, kidney and brain were determined via inductively coupled plasma mass spectrometry (ICP-MS) on a ICAP-QC equipment (ThermoScientific, Waltham, MA, USA). Data analyses were carried out using SPSS Statistics software (version 26). Data are provided as mean values ± standard error of the mean (SEM). Statistical analyses were performed using one-way ANOVA for the liver, feces and urine, and via a non-parametric test for the serum, kidney, and brain. Differences with *p* > 0.05 were considered not significant (ns), * *p* < 0.05, ** *p* < 0.01, and *** *p* < 0.001, n = 6. Graph presentations were drawn with Prism GraphPad 8.0.2.

#### 2.1.2. Quantification of Ceruloplasmin via Tandem Mass Tag (TMT) LC-MS/MS

The quantification of total proteins was carried out in serum samples of 3 mice of each group, using the BCA kit (ThermoScientific, Cat no. 23225). The volume corresponding to 50 μg of protein was taken from each serum sample and diluted to 0.5 mg/mL with cell lysis buffer (Beyotime, Haimen, China, Cat no. P0013G) before trypsinization. The TMTpro16 Labeling Kit (ThermoScientific, Cat no. A44520) was used to label the peptide fragments. LC-MS/MS was performed on these labeled serum samples using a Zorbax Extend-C18 column (2.1 × 150 mm, 5 μm, from Agilent, Santa Clara, CA, USA) at a flow rate of 300 μL/min. The eluent A was H_2_O containing 0.1 vol% of formic acid (FA), and the eluent B was acetonitrile/H_2_O/FA, 80/19.9/0.1, *v*/*v*/*v*). The elution gradient consisted of linear slope segments as follows: from A/B = 98/2 at t_0_ to A/B = 72/28 at 50 min, then A/B = 58/42 at 60 min, then A/B = 10/90 at 65 min. The ratio A/B = 10/90 was then maintained until 75 min. The mass resolution of the first-level MS was set to 60,000, and the maximum injection time was 50 ms. Mass spectrometry scanning was set to the full scan charge-to-mass ratio *m*/*z* range of 350–1500. All MS/MS spectra were collected using high-energy collisional fragmentation in data-dependent positive ion mode. The resolution of MS/MS was set to 30,000 and the maximum ion injection time was 80 ms. The dynamic exclusion time was set to 30 s.

#### 2.1.3. Liver Histology

The mouse liver was fixed in 4% paraformaldehyde solution (Biosharp, Hefei, China) for 24–48 h. After paraffin embedding, serial sections were performed, and each section was 3 μm thick. Paraffin sections were placed in xylene solution twice for 20 min, then placed in absolute ethanol twice for 5 min, and finally placed in 75% ethanol for 5 min; dewaxing was completed after washing. Then, hematoxylin and eosin (HE) stainings were performed using the HE staining kit from Servicebio, China. After that, the sections were dehydrated in anhydrous ethanol 3 times for 5 min, and immersed in xylene twice for 5 min each, to complete the dehydration process. Finally, the slices were examined under a microscope (Nikon Eclipse E100, Tokyo, Japan). The image acquisition was completed in the prime minister system (NIKON DS-U3, Japan).

#### 2.1.4. Activity of Cu,Zn-SOD

The SOD activity was measured using a dedicated Sigma-Aldrich SOD kit. Reagent working solution (WST-1 tetrazolium salt) and enzyme working solution (xanthine oxidase) were prepared according to the supplier instructions. Stock solutions were prepared as follows: bovine Cu,Zn-SOD (Sigma S9697-75KU) at 100 U/mL was dissolved in the “dilution buffer” of the SOD kit, and DPA, TDMQ20 and CuCl_2_ were dissolved in milli-Q water at 10 mM. UV-visible kinetic measurements were carried out on an Agilent Cary 3500 spectrophotometer equipped with magnetic stirring. The reaction mixture was prepared in a UV-visible cuvette, in the following order: dilution buffer (65 µL), 100 units/mL of Cu,Zn-SOD (15 µL) and milli-Q H_2_O (55–100 μL). Eventually, TDMQ20 (300 μM, 45 µL of a 10 mM stock solution) or DPA (50 or 100 μM, 7.5 µL or 15 µL of a 10 mM stock solution) or DPA/CuCl_2_, 1/1, (50 μM, 15 µL of a 5 mM complex solution) was added. The reagent working solution (WST-1, 1.2 mL) and enzyme working solution (xanthine oxidase, 120 µL) were added, and the reaction was immediately monitored at 450 nm (formazan absorbance) for 45 min at 37 °C, under magnetic stirring (800 rpm). The final volume was 1.5 mL. Control experiments without any extra ligand (DPA or TDMQ20) (“fully active SOD” curve) and without Cu,Zn-SOD (“no SOD” curve) were also performed in the same conditions. As an alternative, 100 μM of DPA was added 10 min after starting the measurement (=enzyme working solution “xanthine oxidase” addition).

#### 2.1.5. Reaction of DPA with the Copper Sites of Cu,Zn-SOD

A solution of Cu,Zn-SOD at 10.14 mg/mL was prepared in 0.1 M phosphate buffer, pH 7.4. The theoretical concentration of this solution was [SOD subunit] = [Cu] = 634 μM. Aliquots of a 0.1 M solution of DPA in milli-Q H_2_O were added to reach final concentrations of DPA = 317, 634 and 1268 μM (1.9 μL, 3.8 μL, 7.61 μL), respectively. The total variation in volume in the cuvette was below 1.3 vol% upon the addition of 2 molar equivalent of DPA. The initial UV-visible spectrum of SOD and the spectra of the reaction mixture after the addition of each DPA aliquot were recorded from 200 to 1000 nm.

#### 2.1.6. Reaction of DPA with Vitamin B12

DPA was added to a solution of vitamin B12 in Hepes buffer, pH 7.4. The final concentrations were as follows: [B12] = 20 µM, [DPA] = 2 mM, [Hepes] = 50 mM. Final volume = 1 mL. The mixture was stirred at 37 °C for 1 h and the UV-visible spectrum was then recorded. Control experiments were carried out using B12 alone or DPA alone.

#### 2.1.7. Aerobic Oxidation of Ascorbate Using DPA/Cu^2+^, 1.1/1 or 2.2/1

Buffers and milli-Q H_2_O were treated with Chelex resin (BioRad, Marnes-la-Coquette, France) in order to reduce any traces of metallic ions. A fresh solution of ascorbate was prepared for each oxidation measurement. Typically, solutions of DPA (3 mM, 5.5 µL for 1.1 equivalent or 11 µL for 2.2 equivalent), CuCl_2_ (3 mM, 5 µL) and ascorbate (30 mM, 5 µL) were added to the UV-visible cuvette containing 50 mM of Hepes buffer, pH 7.4, at 25 °C under continuous stirring (800 rpm). The monitoring of the ascorbate absorbance (λ_max_ = 265 nm, ε = 14,500 M*^−^*^1^ x cm*^−^*^1^) [18] was started immediately after ascorbate addition and continued over 30 min. The experiments without ascorbate were also performed in the same conditions and the resulting absorbance was subtracted from measurements with ascorbate (=subtraction of the own copper complex absorbance). The final concentrations in the cuvette were as follows: [Cu] = 10 μM, [DPA] = 11 μM or 22 µM, [ascorbate] = 100 μM, final volume = 1.5 mL. TDMQ20 was also tested as chelator reference.

### 2.2. Animals, Treatments

TX mice were provided by Sun Yat-Sen University (Guangzhou) and C57BL/6 mice by the Guangdong experimental animal center (Guangzhou), and all experiments were performed according to protocols approved by the Welfare Laboratory Animals Committee of the Guangdong University of Technology (approval n°GDUTXS2022083 dated on 3 August 2022), in the framework of the 3Rs principle.

Eight-week-old TX mice (WD) and eight-week-old C57BL/6 mice (Control) were randomly distributed in six groups of 3 males + 3 females, or 2 males + 4 females, each. Each group was then treated using intragastric gavage twice a day (every 12 h) for 14 consecutive days. The TX mice of the WD, TDMQ-L, TDMQ-M, and TDMQ-H groups, respectively, received TDMQ20 doses at 0, 6.25, 12.5 and 25.0 mg/kg, respectively, in NaCl 0.9 wt%. Therefore, the daily doses were 0, 12.5, 25.0, and 50.0 mg/kg/d for the WD, TDMQ-L, TDMQ-M, and TDMQ-H groups, respectively. On the same schedule, C57BL/6 mice received NaCl 0.9 wt%. The DPA group was treated twice a day with DPA at 100 mg/kg in NaCl 0.9 wt% (daily dose = 200 mg/kg/d). The animal groups and treatments are summarized in Table 1.

At the end of the treatment period, the mice (10 weeks old) were placed in metabolic cages for 12 h in order to collect urine and feces. At the end of this 12 h period, the mice were anesthetized using 2.5% Avertin diluted in 0.9% NaCl at a dose of 125 μL/10 g body weight and sacrificed. Cardiac blood was collected and left at room temperature for 30 min before centrifugation at 4 °C at 3000 rpm for 10 min to obtain serum samples. During this 30 min period, the liver, kidney, brain and serum were also collected for copper dosage.

## 3. Results and Discussion

### 3.1. Pharmacological Activity of TDMQ20 on TX Mice

#### 3.1.1. Copper Dosages in TX Mice

The selected drug candidate TDMQ20 has been evaluated on the homozygous toxic milk mouse model (TX model, genetic background C57BL/6) and a regular murine model of Wilson’s disease, suitable for the evaluation of drug candidates [19,20]. TX mice bear a naturally occurring point mutation on ATP7B at position 2135 in exon 8 of the mouse orthologue of human ATP7B, resulting in a methionine change for a valine. The ATP7B gene in TX mice is 82% homologous to that of humans, and TX mice have similar copper biochemical characteristics and pathological processes to those of Wilson’s disease patients, particularly regarding the loss of copper transportation by this protein and the subsequent accumulation of copper in the liver [21].

**Figure 2:**
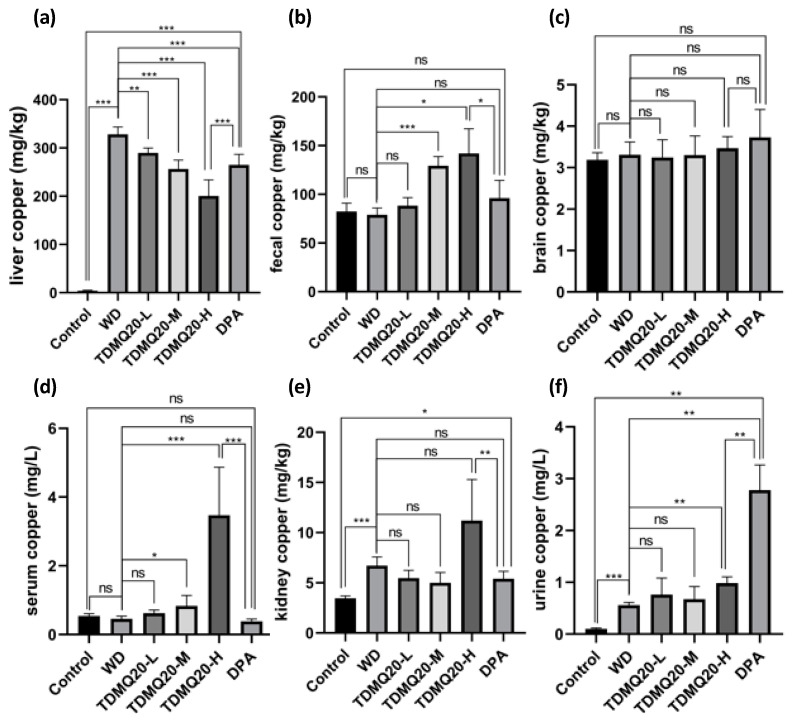
Dosage of copper in liver (**a**), feces (**b**), brain (**c**), serum (**d**), kidneys (**e**) and urine (**f**) of TX mice (WD) after oral treatment using TDMQ20 at 12.5 mg/kg (TDMQ20-L), 25 mg/kg (TDMQ20-M) or 50 mg/kg (TDMQ20-H). WD mice orally treated with DPA at 200 mg/kg are given for comparison. Control mice are healthy C57BL/6 mice bearing no mutation on ATP7B. Differences with *p* > 0.05 were considered not significant (ns), * *p* < 0.05, ** *p* < 0.01, and *** *p* < 0.001, n = 6.

Eight-week-old TX mice (WD) were orally treated using TDMQ20 at daily doses of 12.5, 25.0 and 50.0 mg/kg (in NaCl 0.9%) for 14 days, corresponding to the groups TDMQ-L, TDMQ-M, and TDMQ-H, respectively. As comparison, a group of TX mice was treated with DPA on the same schedule, at a dose of 200 mg/kg/d [22]. The copper concentrations in the liver, kidneys, brain, serum, urine and feces were then determined via ICP-MS, and compared to those of healthy C57BL/6 mice that exhibit no mutation on ATP7B; these were, therefore, used as controls. Individual mouse dosages are reported in Table S1. The mean values calculated for each male or female subgroup clearly indicate that the copper dosages in the mice did not depend on the sex of the animals. Therefore, we considered the mean values of each (M + F) animal group. The results are reported in Table 2 and Figure 2.

First of all, the copper concentration in the liver (Figure 2a) of mice treated using the three different doses of TDMQ20 decreased when the drug dose increased, indicating that the pharmacological effect of TDMQ20 was dose-dependent. At 50 mg/kg/day, TDMQ20 (TDMQ-H) was significantly more efficient in decreasing the copper concentration in liver than D-penicillamine (DPA) at 200 mg/kg/day, which is presently considered the best possible treatment for WD. Noteworthy, the copper concentration in the liver of mice treated at 25 mg/kg/day (TDMQ20-M) was 256 mg/kg, close to the value obtained with DPA at 200 mg/kg/day (265 mg/kg, Table 2). Therefore, the recommended dose of DPA is 8 times higher than that of TDMQ20 for a similar result, indicating a higher efficiency of TDMQ20 compared to DPA. In a correlative manner, the copper content in the feces (Figure 2b) was significantly higher after treatment with 25 or 50 mg/kg/day of TDMQ20, compared to untreated WD mice or to mice treated with DPA at 200 mg/kg/day. This effect, which was also dose-dependent, supports an increased fecal elimination of copper upon treatment via TDMQ20. Therefore, the treatment of the murine model of Wilson’s disease using TDMQ20 resulted in a significant decrease in the copper concentration in the liver, and a significant increase in the fecal excretion of copper compared to untreated mice (indicated as WD mice in tables). This dose-dependent effect indicates that TDMQ20 treatment significantly improves the normal/non-pathological pathway of copper excretion, which is not the case in WD mice treated with the reference drug DPA at 200 mg/kg.

In addition, the copper concentration in the serum of WD mice treated with TDMQ20 increased when the TDMQ20 dose was increased (Figure 2d), confirming the ability of the drug to extract copper from the liver. Conversely, the copper concentration in the serum of WD mice treated with DPA (200 mg/kg/day) was not significantly higher than that of untreated WD mice, and far below that observed for mice treated with TDMQ20 at 50 mg/kg. These data confirmed that TDMQ20 was able to transfer copper to endogenous copper carriers in vivo. The concentration of copper in the kidney of WD mice after treatment with TDMQ20 at 50 mg/kg was higher than that of WD mice by a factor of 1.64 (Figure 2e), similar to the concentration ratio for TDMQ20-H/WD of 1.75 observed in urine (Figure 2f, and data of Table 2). These data indicated an important excretion of copper via the kidney when WD mice are treated with TDMQ20. This excretion mediated by TDMQ20 is also significantly higher than that observed in WD mice treated with DPA at 200 mg/kg.

As expected, the copper concentration in the urine of WD mice was significantly higher than that of the controls (Figure 2f). Upon treatment with TDMQ20 at 50 mg/kg, this was increased by a factor of 1.8 compared to WD mice, indicating a significant improvement in the urinary excretion of copper. Noteworthy, since neurological disorders are often observed in WD patients, it was important to check that TDMQ20 was unable to carry copper to the brain, with putative subsequent redox damage. In fact, the copper concentration was also measured in the brain of mice (Figure 2c). The results indicated that the copper concentration was similar in the brain of the controls and WD mice, and that it was not increased in mice treated with TDMQ20 whatever the drug dose, up to 50 mg/kg/d. Therefore, the transfer of excess copper to the brain mediated by TDMQ20, is unlikely.

#### 3.1.2. Liver Histology

**Figure 3:**
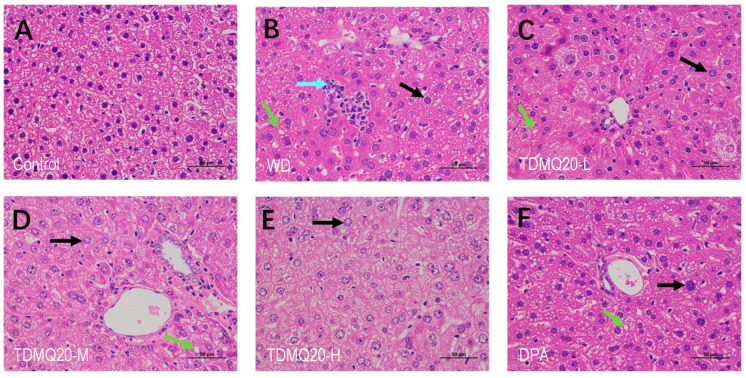
HE staining of representative samples of mouse liver. (**A**) Control group; (**B**) WD group; (**C**) TDMQ20-L group; (**D**) TDMQ20-M group; (**E**) TDMQ20-H group; (**F**) DPA group. Bar scales stand for 50 μm. Black, green and light blue arrows stand for anisonucleosis, steatosis and inflammatory cell infiltration, respectively.

After the hematoxylin and eosin (HE) staining of paraffin-embedded liver sections, 400*×* images of all sections were obtained using an optical microscope. Images of the representative samples are depicted in Figure 3.

The morphology and structure of hepatic lobules in the control group were normal (Figure 3A). The hepatocytes in the WD group (Figure 3B) showed inflammatory cell infiltration (light-blue arrow), extensive steatosis (green arrow), and the anisonucleosis of hepatocytes (black arrow), these two latter features being prominent in WD [23]. It is noteworthy that inflammation and anisonucleosis are related to the oxidative processes [24] that may be induced by liver copper overload. After TDMQ20 oral administration, the liver pathology of the mice treated with low, medium and high doses of TDMQ20 was significantly improved (Figure 3C–E). Compared with the WD group, inflammatory cell infiltrates were no longer detected in the TDMQ20-L and TDMQ20-M groups (Figure 3C,D, respectively), while steatosis decreased in the TDMQ20-M group. The improvement in the high-dose group was the most obvious, with the complete disappearance of cell infiltration and steatosis (TDMQ20-H, 50 mg/kg, Figure 3E). Conversely, the anisonucleosis of hepatocytes (black arrow) and steatosis (green arrow) were still present in liver sections after treatment with DPA at 200 mg/kg (Figure 3F). Therefore, the effect of DPA at 200 mg/kg was similar to that of TDMQ20 at 25 mg/kg, and significantly lower than that of TDMQ20 at 50 mg/kg.

#### 3.1.3. Ceruloplasmin Dosage

In physiological situations, ceruloplasmin (CP) is the major carrier for copper in the blood, accounting for 90% of the circulating copper. The transfer of copper from the liver cytosolic Cu-chaperone, Cu-Atox-1, to ceruloplasmin is mediated by ATP7B. Moreover, apoceruloplasmin in unstable. As a consequence, the ceruloplasmin concentration is typically low in the blood of about half of patients with WD, especially in those with neurological involvement [1].

**Figure 4:**
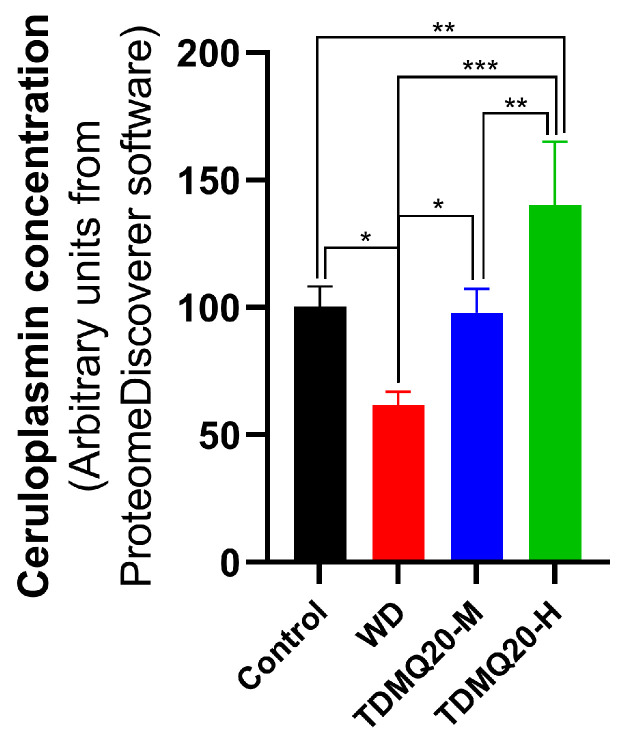
Concentration of serum ceruloplasmin labeled with TMT and detected using LC-MS/MS. * *p* < 0.05, ** *p* < 0.01 and *** *p* < 0.001.

The level of ceruloplasmin was measured using Tandem Mass Tag (TMT) LC-MS/MS in the serum of WD mice. As expected, it was significantly lower than that of the control mice (*p* < 0.05, Figure 4). The treatment with TDMQ20 at 25 or 50 mg/kg/d (TDMQ20-M and TDMQ20-H, respectively), resulted in a significantly increased level of CP in the treated mice, with respect to WD mice, and fully reversed the CP concentration to the level of control. These results were confirmed via the Western blot quantification of serum ceruloplasmin (Supporting Information, Figure S1).

Since the level of CP in serum is considered a significant parameter in the clinical control of Wilson’s disease in humans, the reversal of the CP impairment upon treatment with TDMQ20 confirms that this molecule has a suitable profile as a drug candidate for the treatment of Wilson’s disease.

### 3.2. Compared Behavior of TDMQ20 and DPA towards Cu,Zn-Superoxide Dismutase (Cu,Zn-SOD) and Vitamin-B12

It is noteworthy that DPA is a potent and non-selective ligand for many metal ions with, for example, affinity values as high as log *K*_1_ = 16.5 for Cu(II), log *K*_1_ = 19.5 for Cu(I), and log *β*_2_ = 19.6 for Zn(II) [8]). These affinity values make DPA potentially able to retrieve Cu, Zn or other metal ions from their biological sites and, thus, might impede their function. For example, Cu and Zn are required for the activity of Cu,Zn-SOD, and the affinity of apo-SOD for these two ions is log *K* = 17.2 and 13.4 for Cu(II)and Zn(II), respectively [25], in the same range or below the affinity values of DPA for these metals. Therefore, the potential ability of DPA to inhibit copper and/or zinc enzymes is obviously a matter of concern. DPA also exhibits a significant affinity for Co(II) and Co(III) [8].

Although cobalt is less frequently encountered in metalloenzymes than copper, the cobalt corrin complex vitamin B12 is an important cofactor of several enzymes [26]. Any modification of the stability of vitamin B12 via chelating drugs such as DPA would have a significant impact on metabolism. In fact, the frequent and deleterious effect of B12 deficiency has been reported in the elderly and in neuropathological conditions, including Alzheimer’s disease [27,28,29]. For these reasons, the potential ability of DPA to demetallate vitamin B12 has been evaluated using UV–visible spectrometry, monitoring the absorbance of the cobalt corrin complex at 362 nm.

#### 3.2.1. DPA Inhibits Cu,Zn-SOD While TDMQ20 Does Not

**Figure 5:**
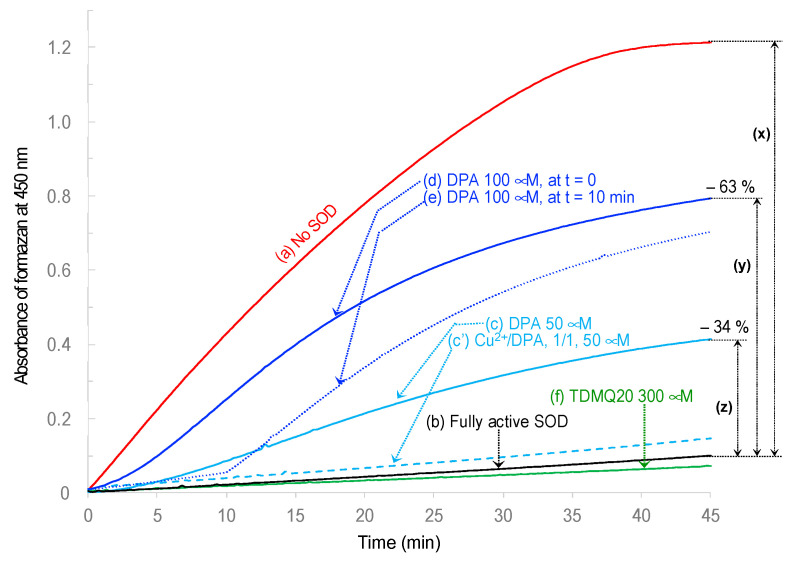
Evaluation of the bovine erythrocyte Cu,Zn-SOD activity (named SOD for short) in the presence of DPA or TDMQ20 added at t_0_ except otherwise stated. Reduction of a tetrazolium salt by O_2_^−•^ produced by xanthine/xanthine oxidase (X/XO) to its formazan derivative (Absorbance at 450 nm): (b) Fully active Cu,Zn-SOD (black trace); (c) in the presence of DPA at 50 μM (light-blue trace); (c′) in the presence of DPA and Cu^2+^, 1/1, 50 μM, preincubated for 2 h at room temperature (light-blue dashed trace); (d) in the presence of DPA at 100 μM (dark-blue trace); (e) in the presence of DPA at 100 μM added at 10 min (dark-blue dotted trace); (f) in the presence of TDMQ20 at 300 μM (green trace). Reduction of tetrazolium in the absence of Cu,Zn-SOD (red trace, (a)) is given as comparison. The percentage of inhibition of Cu,Zn-SOD is calculated as (*y*/*x*) × 100 or (*z*/*x*) × 100 for DPA at 100 μM or 50 μM, respectively (traces d and c, respectively).

Superoxide anion, generated by the xanthine/xanthine oxidase system (X/XO), reduces a specific tetrazolium salt to a formazan dye detected in UV–visible spectrometry, at 450 nm (Figure 5, red trace a). This reaction is fully inhibited by bovine erythrocyte Cu,Zn-SOD that scavenges O_2_^−•^ (Figure 5, black trace b). To evaluate the effect of DPA on SOD activity, DPA was added at t_0_ in the mixture containing X/XO + tetrazolium salt + Cu,Zn-SOD. The production of the formazan dye was inhibited in a DPA dose-dependent manner. In fact, DPA at the final concentration of 50 μM resulted in the significant inhibition of Cu,Zn-SOD (−34%, Figure 5, light-blue full trace c), and DPA at 100 μM resulted in the inhibition of Cu,Zn-SOD by 63% (dark-blue full trace d). Conversely, in agreement with our previous studies [30], adding up to 300 μM of TDMQ20 to the mixture instead of DPA did not induce the significant inhibition of Cu,Zn-SOD (green trace f).

When 100 μM of DPA was added at 10 min, in a mixture containing ongoing active Cu,Zn-SOD (dark-blue dotted trace e), the immediate inhibition of the enzyme was assessed via the rapid increase in the formazan absorbance that was roughly parallel to that of DPA added at t_0_ (dark-blue full trace d).

Owing to the high affinity of DPA for Cu, the inhibition of Cu,Zn-SOD by DPA might be due to the demetallation of the enzyme by DPA, or the denaturation of its copper sites via the formation of a ternary complex between DPA, Cu and the amino acids of the SOD active site. Correlatively, the full activity of Cu,Zn-SOD was retained in the presence of DPA + Cu^2+^ (Figure 5, light-blue dashed trace c’, DPA/Cu molar ratio = 1/1). In fact, the pre-saturation of DPA with copper, whatever the structure of the Cu-DPA complex, prevented the removal of Cu from SOD (or coordination on the Cu site).

**Figure 6:**
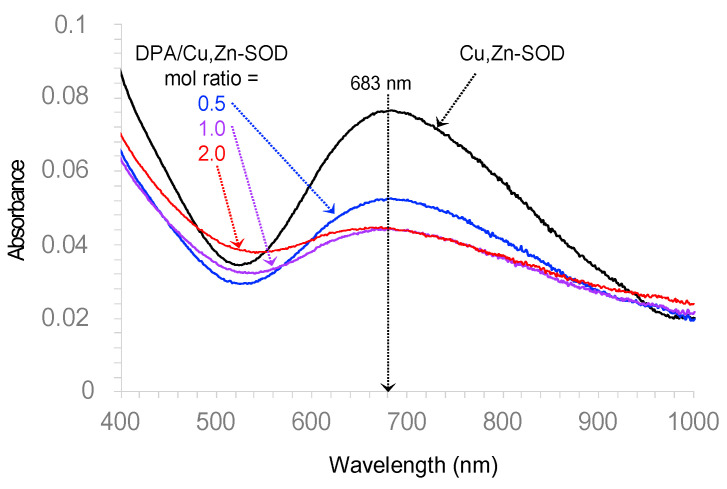
UV–visible spectrum of bovine erythrocyte Cu,Zn-SOD (10.14 mg/mL in phosphate buffer 100 μM, pH 7.4), either alone (black trace) or in the presence of DPA (0.5, 1.0, or 2.0 mol equivalent, blue, violet and red traces, respectively).

To document the ability of DPA to abstract Cu from Cu,Zn-SOD, the UV–visible spectrum of the enzyme was determined either alone or in the presence of DPA, at pH 7.4. The results are depicted in Figure 6. Native Cu,Zn-SOD exhibited an absorption at *λ*_max_ = 683 nm (dark trace), assigned to the d–d transition of Cu(II) complexes with four nitrogen ligands [31,32]. This band drastically decreased in the presence of only 1 molar equivalent of DPA.

These results suggest that the inhibition of Cu,Zn-SOD by DPA probably involves the denaturation of its Cu,Zn active site via a competitive coordination to Cu(II). The inhibition of this vital enzyme for the homeostasis of oxygen metabolism, potentially leading to damaging ROS production, may account for part of the DPA toxicity in clinical use. Conversely, TDMQ20, at the huge concentration of 300 μM, did not interfere with the activity of Cu,Zn-SOD. These results, along with the lack of acute or chronic toxicity of TDMQ20 in mice [16], support the probable absence of deleterious off-target effects of copper chelation caused by TDMQ20, contrary to DPA. TDMQ20 should therefore be considered safer than DPA in vivo.

#### 3.2.2. DPA and TDMQ20 Do Not Demetallate Vitamin B12

**Figure 7:**
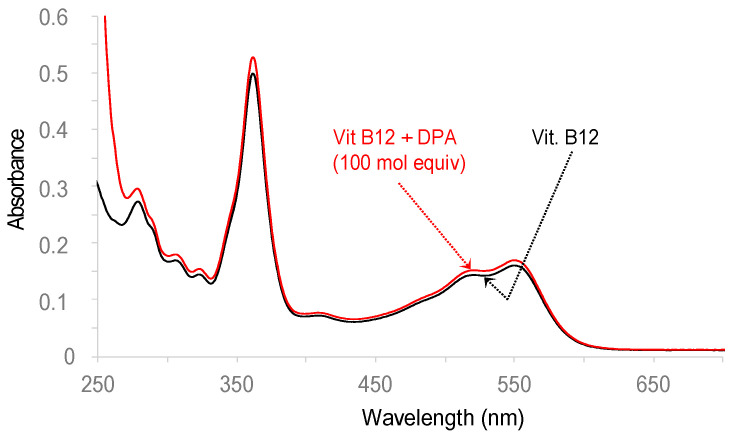
UV–visible spectrum of vitamin B12 alone (20 μM, black trace) or in the presence of DPA (molar ratio = 100/1), in Hepes buffer 50 mM, pH 7.4 (red trace).

Vitamin B12 is a corrinoid cobalt(III) complex involved in folate metabolism, and any event leading to the inactivation of B12 must be avoided. TDMQ20 has already been reported to be inert with respect to vitamin B12, even in large excess, and in the presence of a reducing agent such as glutathione [30]. For comparison, we checked the stability of B12 in the presence of DPA. After incubation for 1 h at 37 °C, the UV–visible spectrum of a mixture of B12 and DPA (1/100 mole ratio) at pH 7.4 (Figure 7, red trace) was the same as that of B12 alone (black trace, *λ*_max_ = 363, 525, 556 nm). This spectrum indicated that DPA was unable to act as a competitive ligand for Co^III^. From this point of view, DPA and TDMQ20 exhibited similar behaviors. In fact, the affinity of Co^III^ and Co^II^ for porphyrins and, even more, for corrins is very high [33] (demetallated only in H_2_SO_4_ 100%, 2 h, 25 °C [34]). Therefore, this enzyme cofactor is not expected to be demetallated under physiological conditions, even in the presence of potent chelators.

### 3.3. The Copper Complex of DPA Triggers the Oxidation of Ascorbate and Production of Reactive Oxygen Species (ROS) While TDMQ20 Does Not

**Figure 8:**
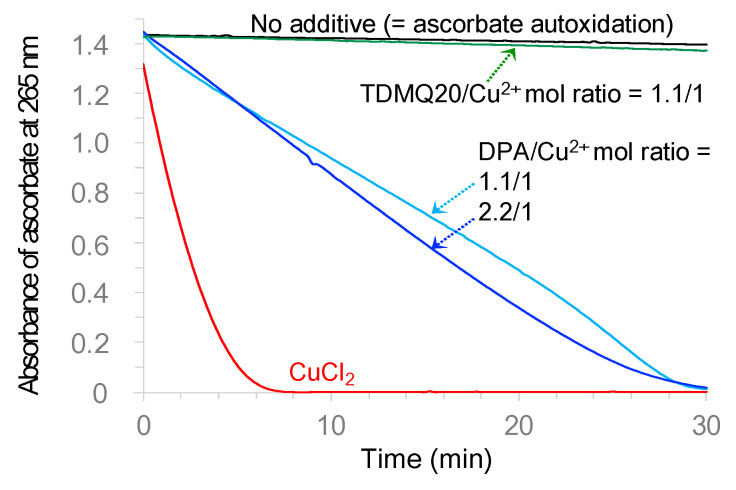
UV–visible (*λ* = 265 nm) kinetic spectra of aerobic ascorbate consumption in the presence of DPA/Cu^2+^, 1.1/1 (light-blue trace), DPA/Cu^2+^, 2.2/1 (dark-blue trace), or TDMQ20/Cu^2+^ 1.1/1 (green trace). Spectra in the presence of no additive (black trace), or CuCl_2_ (red trace) are shown as comparison. Spectra were obtained after subtraction of the absorbances at 265 nm of DPA/Cu^2+^, 1.1/1, DPA/Cu^2+^, 2.2/1, or TDMQ20/Cu^2+^ 1.1/1, respectively, from raw data. [Ascorbate] = 100 μM, [Cu^2+^] = 10 μM, [DPA] = 11 or 22 μM, [TDMQ20] = 11 μM.

The kinetics of ascorbate oxidation in the presence of copper complex(es) of DPA or TDMQ20 were monitored using UV-vis at 265 nm, as a classical test to detect, in vitro, the ROS production induced by Cu(II) in the presence of molecular oxygen [18]. The results are depicted in Figure 8. As expected, the ascorbate was fully oxidized in the presence of CuCl_2_ after a few minutes (<10 min, Figure 8, red trace). In the presence of DPA associated with Cu^2+^, the full oxidation of the ascorbate was achieved in 30 min, regardless of whether the DPA/Cu molar ratio was 1.1/1 or 2.2/1 (light- and dark-blue traces, respectively).

Conversely, consistent with previously reported data [15], the oxidation of the ascorbate in the presence of TDMQ20 associated with Cu^2+^ (TDMQ20/Cu mol ratio = 1.1/1, green trace) was below 4% after 30 min, not significantly different to the ascorbate autoxidation (in the absence of any added copper, black trace). These results suggest that the copper complex of DPA induces oxidative stress in vivo, while TDMQ20 does not.

## 4. Conclusions

TDMQ20 orally administrated at 25–50 mg/kg/day to TX mice decreases the level of copper in liver and favors the excretion of copper in feces, both in a dose-dependent fashion. TDMQ20 is also able to increase the level of ceruloplasmin in serum, a signature of the normal situation. Comparative studies indicate that TDMQ20 is more efficient at such low doses than D-penicillamine at 200 mg/kg/day. Up to the high concentration of 300 μM, TDMQ20 does not interfere in vitro with the activity of Cu,Zn-SOD, contrary to DPA, which was found to significantly inhibit Cu,Zn-SOD at concentrations as low as 50 μM. In addition, the copper complex of DPA produces, in vitro, potentially damaging ROS in the presence of a reducing agent, which is not the case with TDMQ20.

Consequently, TDMQ20 favorably competes with DPA as a potential drug candidate that is able to reverse the accumulation of copper in Wilson’s disease, with fewer side effects than the currently used D-penicillamine drug.

## 5. Patents

A patent application has been filed with the results reported in this article.

## Figures and Tables

**Table 1:** Definition of the six groups of mice and the different drug treatments. Since the gavage was performed twice a day, half of the doses indicated in this table were given at every 12 h.

Mouse Group	Mouse Type	Drug (Daily Dose, mg/kg/d)
Control	C57BL/6	NaCl, 0.9 wt%
WD	TX	NaCl, 0.9 wt%
TDMQ20-L	TX	TDMQ20 (12.5) in NaCl, 0.9 wt%
TDMQ20-M	TX	TDMQ20 (25.0) in NaCl, 0.9 wt%
TDMQ20-H	TX	TDMQ20 (50) in NaCl, 0.9 wt%
DPA	TX	DPA (200) in NaCl, 0.9 wt%

**Table 2:** Concentration of copper in control mice, TX mice (WD), and TX mice treated with TDMQ20 or DPA as mean values for 6 mice of each group ± SEM, calculated using the SPSS Statistics software, v. 26. Results are in mg/kg for solids (liver, feces, kidney and brain) and in mg/L for fluids (urine and serum).

	Cu Content as Mean Value of Six Mice ± SEM Value ^1^					
	Control	WD	TDMQ20-L	TDMQ20-M	TDMQ20-H	DPA
**Liver** (mg/kg)	4.3 ± 0.4	328 ± 6	290 ± 4	256 ± 8	201 ± 14	265 ± 9
**Feces** (mg/kg)	82 ± 4	79 ± 3	88 ± 3	129 ± 4	142 ± 10	96 ± 7
**Serum** (mg/L)	0.54 ± 0.02	0.46 ± 0.03	0.63 ± 0.04	0.83 ± 0.12	3.5 ± 0.6	0.39 ± 0.03
**Kidney** (mg/kg)	3.5 ± 0.1	6.7 ± 0.4	5.5 ± 0.3	5.0 ± 0.4	11.0 ± 1.7	5.4 ± 0.3
**Urine** (mg/L)	0.10 ± 0.01	0.56 ± 0.02	0.76 ± 0.13	0.67 ± 0.10	0.98 ± 0.05	2.8 ± 0.2
**Brain** (mg/kg)	3.2 ± 0.1	3.3 ± 0.1	3.2 ± 0.2	3.3 ± 0.2	3.5 ± 0.1	3.7 ± 0.3

^1^ Individual mouse data are reported as Supporting Information, Table S1. Since, there was no significant sex difference (Table S2), the results reported here are the mean values of six mice (three M + six F, or two M + four F).

## Data Availability

All data can be found either in the article or in Supporting Information published alongside this article.

## Supplementary Materials

The following supporting information can be downloaded at: https://www.mdpi.com/article/10.3390/pharmaceutics15122719/s1, Figure S1: Western blot analysis of serum ceruloplasmin; 30 μg of protein deposited in each lane. (a) Significant example of a gel in the range 70–180 kDa. (b) Densitometric analysis of the serum ceruloplasmin concentration. The result is the mean value (arbitrary units ± SEM) of three independent experiments. * *p* < 0.05, ** *p* < 0.01 and *** *p* < 0.001; *p* values for TDMQ20-M/WD and TDMQ20-M/Control are 0.017 and 0.116, respectively. (c) Images of the complete electrophoresis gels of three independent experiments; Table S1: Dosage of copper in mice; individual raw data; Table S2: Dosage of copper in mice; mean values ± SEM by groups and by sex subgroups. Each group was of n = 6 mice; each subgroup was of n = 3 mice, except otherwise stated. (a) n = 2; (b) n = 4.

## References

[1] Lucena-Valera A., Perez-Palacios D., Muñoz-Hernandez R., Romero-Gómez M., Ampuero J. (2021). Wilson’s disease: Revisiting an old friend. World J. Hepatol..

[2] Forbes J.R., Hsi G., Cox D.W. (1999). Role of the copper-binding domain in the copper transport function of ATP7B, the P-type ATPase defective in Wilson disease. J. Biol. Chem..

[3] Hall A.C., Young B.W., Bremner I. (1979). Intestinal metallothionein and the mutual antagonism between copper and zinc in the rat. J. Inorg. Biochem..

[4] Walshe J.M. (2007). Wilson’s disease. Lancet.

[5] Roberts E.A., Schilsky M.L., American Association for Study of Liver Diseases (AASLD) (2008). Diagnosis and treatment of Wilson disease: An update. Hepatology.

[6] Weiss K.H., Thurik F., Gotthardt D.N., Schäfer M., Teufel U., Wiegand F., Merle U., Ferenci-Foerster D., Maieron A., Stauber R. (2013). Efficacy and safety of oral chelators in treatment of patients with Wilson disease. Clin. Gastroenterol. Hepatol..

[7] Brewer G.J. (2005). Neurologically presenting Wilson’s disease: Epidemiology, pathophysiology and treatment. CNS Drugs.

[8] Martell A.E. (1971). Stability Constants of Metal-Ion Complexes.

[9] Stadtherr L.G., Martin R.B. (1972). Iron(II) and iron(III) complexes of penicillamine. Inorg. Chem..

[10] de Meester P., Hodgson D.J. (1977). Model for the binding of D-penicillamine to metal ions in living systems: Synthesis and structure of L-histidinyl-D-penicillaminatocobalt(III) monohydrate, [Co(L-His)(D-Pen)]·H_2_O. J. Am. Chem. Soc..

[11] Tewari B.B. (2010). Studies on complexation in solution with a paper electrophoretic technique [The system copper(II)/cobalt(II)-methionine-penicillamine]. J. Chem. Eng. Data.

[12] Birker P.J.M.W.L., Freeman H.C. (1977). Structure, properties, and function of a copper(I)-copper(II) complex of D-penicillamine: Pentathallium(I) μ8-chloro-dodeca(D-penicillaminato)-octocuprate(I)hexacuprate(II) n-hydrate. J. Am. Chem. Soc..

[13] Liu Y., Liu X., Huang D., Huang M., Wang D., Nguyen M., Robert A., Meunier M. (2016). New Tetradentate Ligands for Metal Regulation in Neurodegenerative Diseases. Chinese Patent.

[14] Zhang W., Huang D., Huang M., Huang J., Wang D., Liu X., Nguyen M., Vendier L., Mazères S., Robert A. (2018). Preparation of tetradentate copper chelators as potential anti-Alzheimer agents. ChemMedChem.

[15] Zhang W., Liu Y., Hureau C., Robert A., Meunier B. (2018). N4-Tetradentate chelators efficiently regulate copper homeostasis and prevent ROS production induced by copper-amyloid-β_1-16_, even in the presence of an excess of zinc. Chem. Eur. J..

[16] Zhao J., Shi Q., Tian H., Li Y., Liu Y., Xu Z., Robert A., Liu Q., Meunier B. (2021). TDMQ20, a specific copper chelator, reduces memory impairments in Alzheimer’s disease mouse models. ACS Chem. Neurosci..

[17] Huang L., Zeng Y., Li Y., Zhu Y., He Y., Liu Y., Robert A., Meunier B. (2022). Distribution in rat blood and brain of TDMQ20, a copper chelator designed as a drug-candidate for Alzheimer’s disease. Pharmaceutics.

[18] Buettner G.R., Jurkiewicz B.A. (1993). Ascorbate free radical as a marker of oxidative stress: An EPR study. Free Rad. Biol. Med..

[19] Reed E., Lutsenko S., Bandmann O. (2018). Animal models of Wilson disease. J. Neurochem..

[20] Theophilos M.B., Cox D.W., Mercer J.F.B. (1996). The toxic milk mouse is a murine model of Wilson disease. Hum. Molec. Genet..

[21] Voskoboinik I., Greenough M., La Fontaine S., Mercer J.F.B., Kanakaris J. (2001). Functional studies on the Wilson copper P-type ATPase and toxic milk mouse mutant. Biochem. Biophys. Res. Commun..

[22] Zhang J.W., Liu J.X., Hou H.M., Chen D.B., Feng L., Wu C., Wei L.T., Li X.H. (2015). Effects of tetrathiomolybdate and penicillamine on brain hydroxyl radical and free copper levels: A microdialysis study in vivo. Biochem. Biophys. Res. Commun..

[23] Schroeder S.M., Matsukuma K.E., Medici V. (2021). Wilson disease and the differential diagnosis of its hepatic manifestations: A narrative review of clinical, laboratory, and liver histological features. Ann. Transl. Med..

[24] Guzman G., Chennuri R., Voros A., Boumendjel R., Locante A., Patel R., Valyi-Nagy T. (2011). Nucleometric study of anisonucleosis, diabetes and oxidative damage in liver biopsies of orthotopic liver transplant recipients with chronic hepatitis C virus infection. Pathol. Oncol. Res..

[25] Crow J.P., Sampson J.B., Zhuang Y., Thompson J.A., Beckman J.S. (1997). Decreased zinc affinity of amyotrophic lateral sclerosis-associated superoxide dismutase mutants leads to enhanced catalysis of tyrosine nitration by peroxynitrite. J. Neurochem..

[26] Kräutler B. (2005). Vitamin B12: Chemistry and biochemistry. Biochem. Soc. Trans..

[27] Hunt A., Harrington D., Robinson S. (2014). Vitamin B12 deficiency. Br. Med. J..

[28] Weir D.G., Scott J.M. (1999). Brain function in the elderly: Role of vitamin B12 and folate. Br. Med. Bull..

[29] McCaddon A., Regland B., Hudson P., Davies G. (2002). Functional vitamin B(12) deficiency and Alzheimer disease. Neurology.

[30] Huang J., Nguyen M., Liu Y., Robert A., Meunier B. (2019). The TDMQ regulators of copper homeostasis do not disturb the activities of Cu,Zn-SOD, tyrosinase, or the Co^III^ cofactor vitamin B12. Eur. J. Inorg. Chem..

[31] McCord J.M., Fridovitch I. (1969). Superoxide dismutase. An. enzymic function for erythrocuprein (hemocuprein). J. Biol. Chem..

[32] Valentine J.S., de Freitas D.M. (1985). Copper-zinc superoxide dismutase. A unique biological “ligand” for bioinorganic studies. J. Chem. Educ..

[33] Boos R.N., Rosenblum C., Woodbury D.T. (1951). The exchange stability of cobalt in vitamin B12. J. Am. Chem. Soc..

[34] Buchler J.W., Smith K.M. (1975). Static coordination chemistry of metalloporphyrins. Porphyrins and Metalloporphyrins.

