# Supplementary material for: The Specific Copper(II) Chelator TDMQ20 Is Efficient for the Treatment of Wilson’s Disease in Mice
Source: Pharmaceutics. 2023 Dec 2;15(12):2719. doi: 10.3390/pharmaceutics15122719 (PMC10747306; doi:10.3390/pharmaceutics15122719)
Supplement: Supplementary file 1 [file pharmaceutics-15-02719-s001.zip › pharmaceutics-2711239-supplementary.pdf]

Supporting Information

*Western blot dosage of CP in mouse serum***.** Quantification of total proteins was carried out in each serum sample, using the BCA kit (Beyotime, Cat No. P0012S), and each sample was then diluted to 3 mg/mL of proteins. Diluted samples were heated in a water bath at 95°C for 10 minutes to denature proteins. The electrophoresis of proteins was then carried out on commercially available 8% sodium dodecyl sulfate (SDS) polyacrylamide gels (Biosharp, Cat No. BL522A). A constant voltage of 60 V was applied for 30 min, to bring the upper sample proteins to the starting position of the separator gel. Then, a constant voltage of 120 V was applied for 50 min, to separate the proteins according to their molecular weights (the size marker was pre-staining Rainbow Protein Marker 10-180 KDa, ThermoFisher, Cat No. 26617). Under the condition of constant current of 300 A for 50 min, the separated proteins were transfered to a PolyVinyliDene Fluoride (PVDF) membrane, and the PVDF membrane was then blocked with 5% skimmed milk powder for 2 h. Then, the membranes were incubated overnight with the primary antibody at 4 °C (Ceruloplasmin Polyclonal antibody, Proteintech, Cat No. 21131-1-AP, dilution 1/1000 and Transferrin Polyclonal antibody, Proteintech, Cat No. 17435-1-AP, dilution 1/2500). The protein membrane was washed 3 x 10 min with tris-buffered saline containing 0.1% Tween 20 (TBST). The secondary antibody was then incubated at room temperature for 1 h (Affinity brand Goat Anti-Rabbit IgG (H + L) HRP- # S0001, dilution 1/10000). The protein membrane was washed 3 x 10 min with TBST and, then 1 x 5 min with tris-buffered saline (TBS). Finally, the protein membrane was developed with ECL Western blotting substrate (Solarbio, Cat no. PE0010. The experiments were carried out in triplicate.

The equipment used for gel preparation and separation was a Mini-Sub Cell GT Horizontal Electrophoresis system (Bio-Rad, USA) with a DYY-7C Power Supply (Beijing Liuyi Instrument Factory). ECL development was carried out using a Peiqing JS-1070P chemiluminescence gel imaging analyzer (JS-1070P, China).

Figure S1. Western blot analysis of serum ceruloplasmin; 30 μg of protein deposited in each lane. (a) Significant example of a gel in the range 70-180 kDa. (b) Densitometric analysis of the serum ceruloplasmin concentration. The result is the mean value (arbitrary units ± SEM) of three independent experiments. * $p < 0.05$, ** $p < 0.01$ and *** $p < 0.001$; p values for TDMQ20-M/WD and TDMQ20-M/Control are 0.017 and 0.116, respectively. (c) Images of the complete electrophoresis gels of three independent experiments.

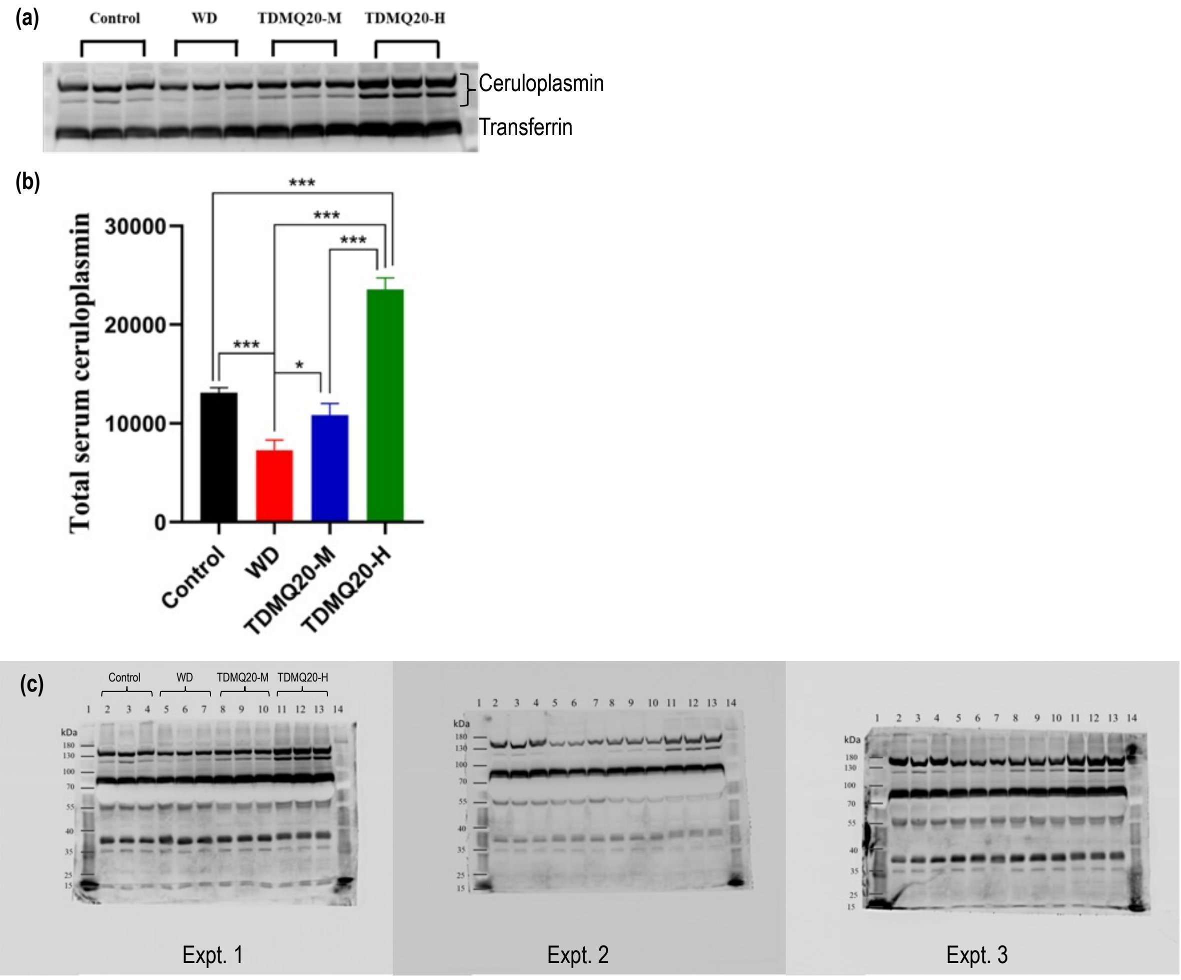

Table S1. Dosage of copper in mice; individual raw data.

Group	mouse sex	mouse no.	liver copper（mg/kg）	kidney copper（mg/kg）	brain copper（mg/kg）	fecal copper（mg/kg）	serum copper（mg/L）	urine copper（mg/L）
Control (C57BL/6)	♂	6	5,42	3,92	3,17	82,2	0,578	0,132
		7	4,77	3,38	2,98	70,6	0,466	0,091
		9	5,12	3,44	3,45	81,1	0,478	0,102
	♀	1	3,58	3,24	3,05	79,9	0,606	0,087
		2	3,23	3,37	3,31	83,6	0,600	0,084
		3	3,39	3,40	3,17	97,1	0,539	0,107
WD	♂	36	345	7,61	3,34	76,7	0,503	0,564
		50	318	6,12	3,79	85,3	0,430	0,521
		51	343	5,64	3,36	85,3	0,331	0,628
	♀	12	325	6,54	3,30	66,3	0,575	0,472
		15	305	7,89	2,82	80,1	0,450	0,577
		41	333	6,45	3,24	79,7	0,458	0,583
TDMQ20-L (12.5 mg/kg/d)	♂	37	285	4,48	3,54	75,9	0,636	0,440
		52	307	5,22	3,19	92,2	0,623	0,420
	♀	16	286	6,56	3,85	94,3	0,673	0,594
		20	283	5,35	3,34	80,0	0,708	1,09
		43	297	5,09	2,82	96,9	0,653	0,899
		44	281	6,17	2,72	89,9	0,454	1,13
TDMQ20 (25 mg/kg/d)	♂	38	274	3,99	3,48	130	0,561	0,483
		53	262	3,80	3,04	123	0,555	0,417
		54	274	4,56	3,05	142	0,683	0,754
	♀	21	247	5,20	3,09	139	1,35	0,650
		24	225	6,14	4,17	123	0,942	0,626
		45	256	6,21	2,97	118	0,911	1,11
TDMQ20-H (50 mg/kg/d)	♂	39	148	7,72	3,77	141	4,60	0,998
		56	239	8,55	3,31	118	3,40	0,798
	♀	26	191	14,9	3,67	118	4,55	1,18
		28	196	12,1	3,26	177	3,88	0,961
		30	195	16,9	3,70	129	3,60	0,973
		47	234	7,09	3,11	168	0,813	0,990
DPA (200 mg/kg/d)	♂	40	226	5,61	3,23	108	0,312	2,65
		57	279	4,29	3,24	122	0,328	2,15
		58	292	4,82	3,32	104	0,401	3,34
	♀	31	267	5,41	3,98	87,3	0,478	2,65
		32	264	6,34	4,97	74,5	0,444	3,38
		34	260	5,94	3,64	80,9	0,352	2,51

Table S2. Dosage of copper in mice; mean values ± SEM by groups and by sex subgroups. Each group was of n = 6 mice; each subgroup was of n = 3 mice, except otherwise stated. (a) n = 2; (b) n = 4.

		Control	**WD**	**TDMQ20-L**	**TDMQ20-M**	**TDMQ20-H**	**DPA**
**Liver (mg/kg)**	**mean(n=6)**	4.3 ± 0.4	328 ± 6	290 ± 4	256 ± 8	201 ± 14	265 ± 9
	**M**	5.1 ± 0.2	335 ± 9	296 ± 11 (a)	270 ± 4	194 ± 45 (a)	266 ± 20
	**F**	3.4 ± 0.1	321 ± 8	287 ± 4 (b)	243 ± 9	204 ± 10 (b)	264 ± 2
**Feces (mg/kg)**	**mean(n=6)**	82 ± 4	79 ± 3	88 ± 3	129 ± 4	142 ± 10	96 ± 7
	**M**	78 ± 4	82 ± 3	84 ± 8 (a)	132 ± 6	130 ± 12 (a)	111 ± 5
	**F**	87 ± 5	75 ± 5	90 ± 4 (b)	127 ± 6	148 ± 14 (b)	81 ± 4
**Serum (mg/L)**	**mean(n=6)**	0.54 ± 0.02	0.46 ± 0.03	0.63 ± 0.04	0.83 ± 0.12	3.5 ± 0.57	0.39 ± 0.03
	**M**	0.51 ± 0.04	0.42 ± 0.05	0.63 ± 0.01 (a)	0.60 ± 0.04	4.0 ± 0.6 (a)	0.35 ± 0.03
	**F**	0.58 ± 0.02	0.49 ± 0.04	0.62 ±0.06 (b)	1.1 ± 0.14	3.2 ± 0.8 (b)	0.42 ± 0.04
**Kidney (mg/kg)**	**mean(n=6)**	3.5 ± 0.1	6.7 ± 0.4	5.5 ± 0.3	5.0 ± 0.4	11 ± 1.7	5.4 ± 0.3
	**M**	3.6 ± 0.2	6.5 ± 0.6	4.9 ± 0.4 (a)	4.1 ± 0.2	8.1 ± 0.4 (a)	4.9 ± 0.4
	**F**	3.3 ± 0.05	7.0 ± 0.5	5.8 ± 0.3 (b)	5.9 ±0.3	12.7 ± 2.1 (b)	5.9 ± 0.3
**Urine (mg/L)**	**mean(n=6)**	0.10 ± 0.01	0.56 ± 0.02	0.76 ± 0.13	0.67 ± 0.10	0.98 ± 0.05	2.78 ± 0.20
	**M**	0.11 ± 0.01	0.57 ± 0.03	0.43 ± 0.01 (a)	0.55 ± 0.10	0.89 ± 0.10 (a)	2.7 ± 0.34
	**F**	0.09 ± 0.01	0.54 ± 0.04	0.93 ± 0.12 (b)	0.80 ± 0.16	1.0 ± 0.05 (b)	2.8 ± 0.27
**Brain (mg/kg)**	**mean(n=6)**	3.2 ± 0.1	3.3 ± 0.1	3.2 ± 0.2	3.3 ± 0.2	3.5 ± 0.1	3.7 ± 0.3
	**M**	3.2 ± 0.1	3.5 ± 0.1	3.4 ± 0.2 (a)	3.2 ± 0.1	3.5 ± 0.2 (a)	3.3 ± 0.03
	**F**	3.2 ± 0.1	3.1 ± 0.2	3.2 ± 0.3 (b)	3.4 ± 0.4	3.4 ± 0.1 (b)	4.2 ± 0.4
